# 

**Published:** 1896-08

**Authors:** 


### Communicated.

NEWARK, N. J. August 1, 1896.

EDITOR THE AMERICAN JOURNAL OF DENTAL SCIENCE:

At the twenty-sixth annual meeting of the New Jersey State Denial Society, held at Asbury Park, N. J., July 29th to 31st, inclusive, the following named officers were elected:

President, Harvey Iredell, D. D. S., New Brunswick, N. J.; Vice-President, J. L. Crater, D. D. S., Orange, N. J.; Secretary, Charles A. Meeker, D. D. S., Newark, N. J.; Treasurer, Geo. C. Brown, D. D. S., Elizabeth, N. J.

CHAS. A. MEEKER, Secy.

### To Readers and Correspondents.

Communications intended for publication, and exchange journals and papers, should be addressed to the Editor of The American Journal of Dental Science, No. 845 N. Eutaw St. Baltimore, Md. All letters relating to the business of the Journal, or containing cash remittances, to be directed to Snowden & Cowman Mfg. Co., 9 W. Fayette Street, Baltimore, Md. Articles for publication should be received by the Editor at least two weeks previous to the first of the month on which the number in which it is designed for them to appear is issued.

☞ The American Journal of Dental Science will contain fifty pages of reading matter in each number, making a volume of about Six Hundred pages,

EXCLUSIVE OF ADVERTISEMENTS.

